# Effects of ethnicity and vitamin D supplementation on vitamin D status and changes in bone mineral content in infants

**DOI:** 10.1186/1471-2431-12-6

**Published:** 2012-01-16

**Authors:** Steven A Abrams, Keli M Hawthorne, Stefanie P Rogers, Penni D Hicks, Thomas O Carpenter

**Affiliations:** 1 Department of Pediatrics, Baylor College of Medicine and Texas Children's Hospital, Houston, Texas 77030, USA; 2 Departments of Pediatrics (Endocrinology) and Orthopedics and Rehabilitation, Yale University School of Medicine, New Haven, CT 06511, USA

**Keywords:** breastfeeding, vitamin D, bone mineral content

## Abstract

**Abstract:**

**Background**

To evaluate the effects on serum 25(OH)D and bone mineralization of supplementation of breast-fed Hispanic and non-Hispanic Caucasian infants with vitamin D in infants in Houston, Texas.

**Methods**

We measured cord serum 25(OH)D levels, bone mineral content (BMC), bone mineral density (BMD) and their changes over 3 months of life with 400 IU/day of vitamin D3 supplementation.

**Results**

Cord serum 25(OH)D was significantly lower in Hispanic than non-Hispanic Caucasian infants (16.4 ± 6.5 ng/mL, n = 27, vs 22.3 ± 9.4 n = 22, p = 0.013). Among 38 infants who completed a 3 month vitamin D supplementation intervention, provision of 400 IU/day of vitamin D increased final 25(OH)D to a higher level in non-Hispanic Caucasian compared to Hispanic infants. There was no significant relationship between cord serum 25(OH)D and BMC or BMD in the first week of life (n = 49) or after 3 months of vitamin D supplementation.

**Conclusion**

Low cord 25(OH)D levels are seen in Hispanic infants, but their functional significance is uncertain related to bone health in a southern US setting. Daily vitamin D intake of 400 IU during the first months of life appears adequate to increase serum 25(OH)D and support BMC increases despite low initial 25(OH)D levels in some infants.

**Trial Registration:**

ClincalTrials.gov NCT00697294

## Background

Vitamin D has been given to infants to prevent and treat rickets for almost 100 years. The recommended dose of 400 IU (10 micrograms) daily for infants was established based on typical amounts in a teaspoon of cod liver oil [1]. This recommendation has recently been reaffirmed as being appropriate by both the Institute of Medicine [2] and the American Academy of Pediatrics [3] whereas the Endocrine Society considers the 400 IU to be a minimum appropriate dose [4]. Although the dose of 400 IU daily is used for both breast-fed and formula-fed babies, vitamin D-deficient rickets is extremely rare in formula-fed infants primarily due to the mandatory addition of vitamin D to infant formulas in the United States. Formula-fed infants typically consume 300-500 IU/day from their formula and therefore generally meet the current recommendations without an additional oral supplement of vitamin D drops especially after the first 6 to 8 weeks of life. The relatively greater calcium and phosphorus concentration in US infant formulas compared to human milk may also be protective against rickets in some infants.

Remarkably, few data have looked at modern techniques of assessing bone mineral status and compared it to vitamin D levels and supplementation in this population [5]. Serum 25-hydroxyvitamin D (25(OH)D) is accepted as the best marker of vitamin D status although it may serve primarily as a marker of exposure rather than clinical outcomes [2].There are few data that correlate serum 25(OH)D levels with functional outcomes in newborns and small infants.

Neonatal serum 25(OH)D levels reflect a range of *in utero *factors, most significant of these being the maternal vitamin D status [6]. Although calcium and phosphorus are transported transplacentally without vitamin D, the effects of low vitamin D *in utero *or shortly after birth are uncertain [7].

This study was conducted to evaluate early life bone mineral content (BMC) and the effects of three months of vitamin D supplementation in breast-fed Hispanic and non-Hispanic Caucasian (henceforth, "Caucasian") infants on vitamin D levels. We hypothesized that 25(OH)D would be lower in Hispanic than Caucasian newborns but that these differences would not be substantial enough to be related closely to BMC and bone mineral density (BMD) or their changes during the first three months of life.

Due to the guidelines for providing vitamin D supplements to all breast-fed infants, it is not ethically possible to conduct a placebo-controlled study of vitamin D supplementation in the United States. However, the relationship between vitamin D status and bone-related outcomes in supplemented infants provides information about the effects of these cord levels and vitamin D supplementation in early infancy. Furthermore, few data have evaluated Hispanic infants who may be at high-risk for low vitamin D status due to poor maternal vitamin D intake [4].

## Methods

### Subjects

Subjects were recruited from two hospitals in the Texas Medical Center in Houston, Texas. The latitude of Houston, TX is 29 degrees, more southern than the typically used latitude cutoff of Atlanta, Georgia of 33 degrees for sunlight exposure to lead to vitamin D formation in the dermis to occur throughout the year. The two hospitals consisted of a private hospital from which most of the Caucasian subjects were recruited (St. Luke's Episcopal Hospital) and a nearby public hospital (Ben Taub General Hospital) from which nearly all of the Hispanic patients were recruited. Subjects were enrolled whose mother was healthy (non insulin-dependent diabetic, no other major pregnancy complications), who were expected to have a singleton, non-small for dates infant, and had been admitted to the labor and delivery unit due to the onset of labor or were admitted for a planned induction or c-section delivery at 37 to 41 weeks gestation. Consent was obtained prior to delivery. Approval was obtained from the Institutional Review Board of Baylor College of Medicine and Affiliated Hospitals.

### Procedures

Cord blood was obtained at birth for analysis of 25(OH)D and ionized calcium. One week later, subjects returned to the research facility for whole body dual-energy x-ray absorptiometry (DXA) measurements and to receive the vitamin D supplements (400 IU/mL) with instructions to give daily by mouth until the final study visit. Subjects who attended this visit were considered enrolled. Mothers were instructed to continue the supplements as long as they were breastfeeding. If a mother weaned her child to an exclusively formula-fed diet, she was instructed to discontinue the supplements but to return for the final study visit regardless. A brief questionnaire on prenatal vitamins, including name and frequency, and intake of vitamin D rich foods was completed. The Muslim community in Houston represents about 3% of the population, and therefore, mothers were asked if they had any religious or cultural practices that included covering their head and bodies with clothing in the context that this may affect sunlight exposure and therefore cord 25(OHD) values. Other lifestyle and dress habits were not evaluated, and all participants were observed by the study staff for typical Western-style US clothing (sleeveless or short-sleeved shirts in the summer, jackets or coats in the winter).

The final study visit was conducted at three months of age. Subjects returned for a repeat DXA scan and a serum sample was obtained for 25(OH)D and parathyroid hormone (PTH) levels. Mothers were asked to return the supplements bottles at the final visit. The weight of the supplements bottles at this visit compared to when they were given to the mothers at the first visit was used as a measure of compliance of how often the drops were given to the infant. Birth weight and gestational age were recorded for each infant. Anthropometrics were recorded at each outpatient visit using a digital scale and length board. Cord blood and infant blood at three months of age were analyzed for 25(OH)D concentration by DiaSorin radioimmunoassay (DiaSorin Inc., Stillwater MN) at Yale University. Results of samples analyzed in this assay are consistently found to agree with the mid-range of outcomes of those using this assay and participating in the international DEQUAS standardization system. The inter- and intra-assay coefficients of variation in that laboratory are 9.6% and 6.6%, respectively. Ionized calcium was measured from cord blood using standard clinical measurement techniques in the blood gas laboratories of the respective hospitals. Intact PTH was measured by immunochemiluminometric assay (ICNA) with a sensitivity of 3 pg/mL. Throughout the manuscript, we have provided values for 25(OH)D as ng/mL, these may be multiplied by 2.5 to obtain results in nmol/L.

Whole body DXA measurements, BMC, and BMD were conducted using Hologic Delphi Model (Hologic Inc., Waltham MA). Reproducibility of DXA Infant Scans; BMC = 3.8% and BMD = 4.1%. The DXA instrument undergoes regularly scheduled quality control testing for phantom reproducibility and signal uniformity. Subject scanning does not take place unless all quality control results fall within acceptable limits. Serum 25-OHD was measured by radioimmunoassay kit methodology (DiaSorin, Stillwater, MN).

### Statistical Analysis

Sample size determination (at least 16 each Hispanic and Caucasian) was done to identify a one standard deviation difference in the change in bone mineralization between one week of age and three months of age by ethnicity. Differences markedly less than are less likely to be biologically relevant and this study was not designed to identify small differences.

Comparisons of values between study time points were made using a generalized linear regression model with covariate adjustment based on the specific analysis conducted. Analysis was performed using SPSS 18.0 for Macintosh (SPSS, Inc., Chicago, IL). Simple and multiple regression analysis were used as appropriate. Significance was assumed at a p < 0.05. All data are presented as the mean ± SD except as noted. Statistical analysis was not applied to the relative change in serum 25(OH)D values with low baseline versus high baseline data as there is currently no accepted optimal approach for such an evaluation as reviewed recently by Tu and Gilthorpe [8]. To convert serum vitamin D levels from ng/mL to nmol/L, multiply by 2.5.

## Results

### Cord blood and initial bone mineralization values

A total of 49 singleton infants were enrolled at one week of age for this research project. Of these, 38 (78%) returned at three months for the final study visit. There was no significant effect of birth hospital on the serum 25(OH)D level when ethnicity was accounted for by covariate analysis (p = 0.37).

Of the 49 study subjects, 22 were Caucasian and 27 Hispanic. The mean serum cord 25(OH)D values for each group were 22.3 ± 9.4 ng/mL for Caucasian and 16.4 ± 6.5 ng/mL for Hispanic infants (p = 0.013 for difference). We evaluated the relationship between cord blood 25(OH)D and BMC and BMD at one week of age. Cord 25(OH)D was not significantly related to BMC (p = 0.39) or BMD (also, p = 0.39). BMC was highly correlated to body weight (r = 0.86, p < 0.001) as was BMD (r = 0.56, p < 0.001). BMC was significantly related to length at one week (r = 0.61, p < 0.001) however, in multivariate analysis including weight and length, only weight was significant, p < 0.001 and length was not significant, p = 0.87. Length was similar between Hispanic and Caucasians, 50.3 ± 2.1 cm versus 50.5 ± 2.0 cm respectively, p = 0.72. Weight was also not significantly different between groups at this time, 3.68 ± 0.4 kg versus 3.47 ± 0.4 kg for Hispanic and Caucasians respectively, p = 0.08.

Cord 25(OH)D averaged 14.9 ng/mL and BMC at one week of life averaged 70.3 g for the 2 Caucasian infants who did not return at 3 months. Cord 25(OH)D averaged 16.3 ng/mL and BMC at one week of life averaged 72.6 g for the 8 Hispanic infants who did not return at 3 months.

Two mothers (1 Hispanic, 1 Caucasian) reported covering their face and body due to their religious beliefs. No other clothing or lifestyle habits affecting sunlight exposure were observed by the study staff as related to ethnicity. All mothers reported taking a prenatal vitamin supplement throughout the pregnancy. None of the mothers reported taking additional vitamin D supplements separate from their prenatal vitamins. Prenatal vitamin supplement composition varied by brand; however, most brands included 200 IU vitamin D per pill. Average maternal intake of vitamin D from foods and prenatal supplements was 439 ± 94 IU/day.

### Results at 3 months

Overall compliance with the vitamin D drops was excellent with 90% of the drops given overall based on measurement of the volume of returned droppers. There was no relationship between compliance and outcome so this was not further considered in the analysis. Overall, 47% of the infants (18/39) were exclusively breast-fed at 3 months, 28% primarily breast-fed (51-90% human milk) and 25% primarily formula-fed. Results were not related to feeding type.

**Figure 1:**
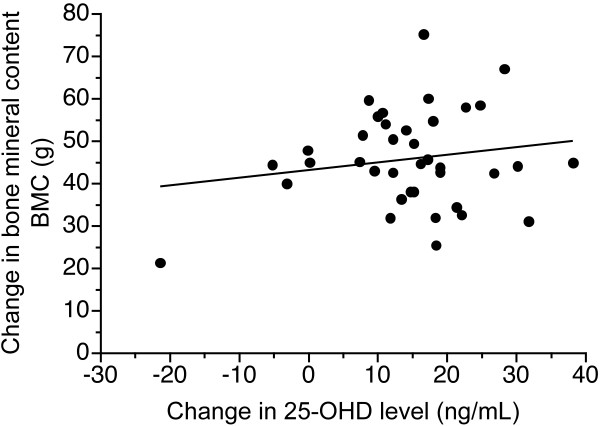
**Relationship between changes in 25(OH)D level during 3 months of supplementation and change in total body bone mineral content, r = 0.17, p = 0.30, effect size = 0.18**. Note that values in ng/mL can be converted to nmol/L by multiplying by 2.5.

Results for the 38 infants (19 Hispanic and 19 Caucasian), with the values obtained initially, are shown in Table 1. Of note is that the BMC and BMD were greater in Hispanic infants at three months but not at one week of age. When looking at the change in BMC during the study period, the change was significantly greater in Hispanic infants than Caucasians (51.0 ± 11.3 g vs 41.2 ± 10.1 g, p = 0.006). For BMD, changes were greater in Hispanic than Caucasians (0.019 ± 0.012 g/cm^2 ^vs 0.010 ± 0.012 g/cm^2^, p = 0.017). When body weight at three months was included as a covariate in this analysis, changes in BMC remained significant, p = 0.03, but changes in BMD were not (p = 0.06). Overall, there was no relationship between changes in 25(OH)D and BMC (Figure 1).

**Table 1:** Results from 38 subjects who completed 3 month supplementation study

	Non-Hispanic Caucasian (n = 19)	Hispanic (n = 19)	p-value
Cord 25(OH)D (ng/mL)	23.0 ± 9.4	16.9 ± 7.2	0.03
BMC at one week (g)	69.4 ± 9.1	72.8 ± 9.2	0.27
BMD at one week (g/cm2)	0.196 ± 0.010	0.199 ± 0.011	0.29
3 month BMC (g)	110.6 ± 13.9	123.8 ± 13.4	0.005
3 month PTH (ng/L)	14.4 ± 16.4 (n = 19)	17.6 ± 10.5 (n = 18)	0.48
3 month total serum Ca (mg/dL)	10.6 ± 0.4 (n = 19)	10.6 ± 0.4 (n = 17)	0.70
3 month BMD (g/cm2)	0.205 ± 0.013	0.219 ± 0.012	0.002
3 month weight (kg)	6.0 ± 5.8	6.4 ± 6.1	0.10
3 month length (cm)	60.6 ± 2.2	61.2 ± 2.7	0.41
3 month 25(OH)D (ng/mL)	37.7 ± 7.6	31.2 ± 8.1	0.014

Data are Mean ± SD.

BMC = bone mineral content

BMD = bone mineral density

PTH = serum parathyroid hormone

25(OH)D = serum 25-hydroxyvitamin D concentration (conversion to nmol/L is by multiplying values shown by 2.5)

For entire 49 subjects enrolled: Values for cord 25(OH)D, Non-Hispanic Caucasian (n = 22), 22.3 ± 9.4 ng/mL and Hispanic (n = 27), 16.4 ± 6.5 ng/mL, p = 0.01. Values for BMC at one week, Non-Hispanic Caucasians, (n = 22), 69.5+8.8 (g) and Hispanic, (n = 27), 72.4 ± 9.2 (g), p = 0.27. Note that values in ng/mL can be converted to nmol/L by multiplying by 2.5.

We further evaluated the relationship between cord 25(OH)D and outcomes using a general linear regression model. For this model, gender, birth weight, season of measurement and ethnicity were covariates. There was no significant effect of the percent of human milk provided on outcomes including change in BMC (p = 0.89) or change in 25(OH)D (p = 0.25) so all data were analyzed without amount of human milk included as a covariate. We found that cord 25(OH)D (dependent variable) was not significantly related to BMC and BMD as shown before, and was not closely related to BMC at three months (p = 0.10). Cord and 3-month 25(OH)D levels were not significantly related to weight, length or head circumference at 3 months. In addition, 3-month 25(OH)D levels were not closely related to BMC at 3 months, r = 0.22, p = 0.18.

**Figure 2:**
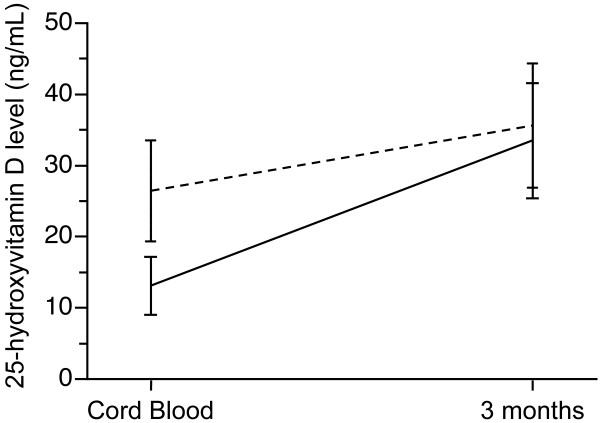
**Baseline and final values for 25(OH)D in 38 subjects based on initial values above or below 20 ng/mL**. Note that values in ng/mL can be converted to nmol/L by multiplying by 2.5.

We considered infants with cord 25(OH)D below 20 ng/mL compared to those above 20 ng/mL regardless of ethnicity. As shown in Figure 2, there was a greater increase in 25(OH)D in those with starting values < 20 ng/mL (n = 18, increase of 20.4 ± 8.2 ng/mL) versus an increase of 9.2 ± 10.6 ng/mL in those 20 subjects with a starting 25(OH)D value > 20 ng/mL (Figure 2).

## Discussion

We found 25(OH)D levels < 20 ng/mL to be common in the cord blood of infants in a southern climate in the United States representing about 60% of the infants in our study, with about 20% of the infants having values ≤ 10 ng/mL. However, we did not identify any specific physiological consequences of these cord 25(OH)D levels for bone mineralization at birth as evidenced by initial DXA measurements. Supplementation with 400 IU/day of vitamin D to these infants led to a relatively greater increase in those who were born with the lowest vitamin D status and there was no suggestion of any bone mineral outcome deficits at one week or three months of age in infants with low cord 25(OH)D levels. We also did not find any significant relationship between cord 25(OH)D values and growth outcomes. We note that our study was of limited size however, and that larger studies are needed in diverse global populations. Our study did not have the ability to identify the effects of small amounts of formula or other nutritional differences between groups.

The close relationships between weight and length and initial BMC imply that calcium transfer across the placenta is closely related to overall nutrient transfer and not to vitamin D status (7). Of note is that few subjects had a cord 25(OH)D < 6 ng/mL, a value found in studies in pregnant women and newborns in the Middle East [9,10]. Neonatal rickets and hypocalcemia have been reported in some, but not all infants with cord or maternal 25(OH)D values < 6-10 ng/mL from the Middle East. These extremely low values are not commonly reported in the United States, although we speculate that an increasing number of cases may be reported in the future due to increased awareness of vitamin D deficiency.

There are very few data on 25(OH)D in cord blood in the Hispanic population and no data looking specifically at the relative increase over time based on cord 25(OH)D [11]. We did not have PTH values on the cord blood but PTH values at three months of age were not correlated significantly with 25(OH)D values at birth or three months or with bone mineral outcomes. Differences in 25(OH)D between Hispanic and Caucasian infants may be related to lifestyle including sunshine exposure. These were not evaluated in this study but there are no clear cultural reasons to expect a substantial difference in Houston.

In a European population, 64% of infants had a serum 25(OH)D at 3-6 days of age that was below 12 ng/mL [12]. Of note is that in that study, about 10% of infants with these very low 25(OH)D levels had evidence of hypocalcemia although none had physical symptoms and it is not clear if there was a true cause and effect in this group. Although there was a trend in that study towards a relative increase in 25(OH)D in infants with lower baseline values who received supplementation, this was not clearly demonstrated. In the United States, median values for 25(OH)D in a northern setting in the first days of life were 17 ng/mL with 58% of infants < 20 ng/mL, results very similar to those seen in our study [13].

We found similar ionized calcium levels in Hispanic and Caucasians at birth. There is some suggestion that late hypocalcemic tetany is more common in Hispanic infants, although this is not well demonstrated due to the limited published data [14]. Regardless, serum calcium in the cord blood and in the first days of life is likely controlled by a variety of factors, of which vitamin D is only one factor.

Limited previous research is generally consistent with our findings. Park et al [15] studied Korean infants at 2 to 5 months of age and found no relationship between 25(OH)D level and BMC assessed by DXA even though many of the infants had very low 25(OH)D levels. They speculated that this is due primarily to passive absorption of calcium at this age. It has been suggested but is not proven that early life calcium absorption is primarily non-vitamin D dependent [2]. However, it is likely that by three months of age, vitamin D has a key role in calcium absorption, especially in breast-fed infants with a relatively low calcium intake compared to formula-fed infants. Whether the greater bioavailability of calcium from human milk compared to formula affects the needed level of 25(OH)D by infants is unknown.

Greer et al [16] found an increase in 25(OH)D from 24 to 39 ng/mL at three months of age with the provision of 400 IU/day of vitamin D to breast-fed infants. These results are similar to ours, with slightly higher 25(OH)D values found in the Greer study. Direct comparisons however of 25(OH)D values between studies conducted in the past and recent studies should be done with caution due to well-documented variations in assay techniques.

Therefore, in considering early vitamin D supplementation, the question becomes whether there is physiological benefit to rapid replenishment with vitamin D (e.g. high doses of vitamin D in early life) with or without monitoring of 25(OH)D levels or whether the 400 IU/day dose is adequate. Our data, from this small dataset, can only be used to partially answer this question specifically related to bone health. However, it appears that, at least in a southern US setting, 400 IU/day is adequate for infants regardless of vitamin D status at birth. We cannot rule out the possibility that a small number of infants will be hypocalcemic in the first weeks of life with this approach, but, this appears to be uncommon and may be more related to PTH function than vitamin D. Further research is needed relative to the etiology of symptomatic hypocalcemia in the first weeks of life.

Studies in adults have found a greater increase in 25(OH)D levels in subjects who started at a lower level before supplementation [17]. Similar data are not readily available in pediatric populations. Caution should be used in interpreting any results for changes in 25(OH)D based on baseline levels. It has been suggested that these results may reflect a regression to the mean phenomenon or may be related in part to measurement variability (8). However, the findings in our study appear meaningful in that the provision of vitamin D at a dose of 400 IU/day led to mean values that were above 30 ng/mL regardless of starting value or ethnicity. The 30 ng/mL target is well above that likely to be needed for adequate vitamin D status in the newborn based upon the recent recommendations of the Institute of Medicine [2].

The effects of maternal vitamin D deficiency are complex and may extend beyond bone health effects in infants. Provision of all pregnant and lactating women with the Recommended Daily Allowance of 600 IU/day is an important public health strategy. Nonetheless, such supplements are not universally consumed by the population. The Hispanic community may be at higher risk due to darker skin pigmentation, greater obesity, lower dairy intake and less supplement use. It has been suggested that all Hispanics and all pregnant and lactating women have their 25(OH)D levels monitored [4].

Of note is that 25(OH)D levels in a southern United States climate are low despite sunshine exposure year round at the latitude in Houston. Furthermore, there is a seasonal dependence of 25(OH)D levels although we did not have enough subjects to identify the degree to which this seasonal dependence was affected by ethnicity. We had previously shown a seasonal dependence of 25(OH)D levels in prepubertal girls in Houston [18] with no differences in calcium absorption or kinetics between Hispanic and Caucasian prepubertal girls. Thus, it is clear that a southern US location is not protective against seasonal deficiency of vitamin D or low cord 25(OH)D levels.

## Conclusion

We found that low 25(OH)D levels are common in newborns in a southern US climate, especially among Hispanic infants. Improvement occurred with vitamin D supplementation and was not related to changes in bone mineral content. Our findings support current guidelines to begin vitamin D supplementation of 400 IU/day to all breast-fed infants in the first week of life and to further encourage the provision of adequate vitamin D to pregnant women. Larger studies are needed in diverse populations at various latitudes to further characterize the relationship between vitamin D supplementation and bone mineral content.

## Abbreviations

25(OH)D: 25-hydroxyvitamin D; BMC: Bone Mineral Content; BMD: Bone Mineral Density; DXA: dual-energy x-ray absorptiometry.

## Competing interests

The authors declare that they have no competing interests.

## Authors' contributions

Each author was involved in all aspects of study design, data interpretation and manuscript preparation. All authors read and approved the final manuscript.

## Pre-publication history

The pre-publication history for this paper can be accessed here:

http://www.biomedcentral.com/1471-2431/12/6/prepub
